# Price, internet penetration and green food industry development: Based on the interaction between demand and supply

**DOI:** 10.1371/journal.pone.0289843

**Published:** 2023-09-08

**Authors:** Yongqiang Zhang, Pengju Wan, Guifang Ma, Paola Andrea Pereira Uñate

**Affiliations:** 1 College of Economics and Management, Northeast Agricultural University, Harbin, Heilongjiang, China; 2 School of Automotive and Mechatronics Engineering, Harbin Cambridge University, Harbin, Heilongjiang, China; Universidad Nacional Autonoma de Nicaragua Leon, NICARAGUA

## Abstract

The development of the green food industry can not only meet people’s demand for high-quality food and promote the sustainable development of the ecological environment but also carry the additional expectation of realizing rural revitalization. Based on the data of Heilongjiang province from 2000–2021, we examined the dynamic effects of price fluctuations and Internet penetration on the green food industry using a system dynamics model. The empirical results showed that both price fluctuations and Internet penetration affect people’s demand for green food, which in turn affects the development of the green food industry. The inhibitory effect of price fluctuation on green food industry is more obvious in the early stage of green food industry development, and Internet penetration always significantly promotes the development of green food industry. Moreover, the Internet penetration can effectively mitigate the negative impact of price fluctuation on the green food industry, and the impact becomes more significant with the increase of Internet penetration. The results of this study can help promote the sustainable development of the green food industry.

## Introduction

In contemporary times, the matters of health and the environment have attained paramount significance within the international community. The Food and Agriculture Organization (FAO) has fostered extensive collaborations with nations to conceive and implement the "One Health" strategy, aiming to enhance worldwide food safety measures and uphold the well-being of consumers. Food is widely recognized as one of the foremost contributors to environmental impact among the three key consumption domains. The indiscriminate use of chemicals by individuals has resulted in severe environmental degradation, which has consequently drawn significant attention to the concept of green food [1]. The ascent and evolution of the green food industry hold the potential to exert a profound impact on various critical aspects, including safeguarding the ecological environment, enhancing the quality of agricultural products, fostering industrial development, and preserving human health.

Currently, China’s green food industry has made notable strides in its development, characterized by the continual expansion of green food production scale, the steady enhancement of quality standards, and the burgeoning prominence of brand advantages. At present, China boasts a diverse range of green food categories, comprising five principal categories and 57 sub-categories. According to statistical data up to the year 2020, China is home to nearly 20,000 green food enterprises, collectively offering an impressive array of over 40,000 distinct product types. The swift progress of the green food industry in China has resulted in a persistent reduction in the utilization of chemical fertilizers in agriculture. This positive trend has provided significant impetus for the cause of ecological environmental protection. From a demand perspective, there has been a noteworthy shift in people’s consumption patterns, driven by a deepening concern for sustainable development. This shift is indicative of a discernible trend towards consumption upgrading [2]. A report titled "Wellness Worldwide: Consumer Insights from Four Countries," published by McKinsey & Company in 2021, highlights the growing preference among consumers for personalization, along with an increasing demand for natural or "clean" products [3]. Furthermore, the resilience exhibited by green food sales in sustaining growth, even in the face of the challenges imposed by the COVID-19 pandemic, is a testament to the burgeoning prominence of the notion of sustainable consumption [4]. Of particular significance is the observation that Chinese consumers exhibit a greater inclination towards personalizing consumer products, surpassing even their counterparts in developed nations. Additionally, they demonstrate a pronounced preference for purchasing green foods, primarily driven by health-related considerations [5, 6]. The China Green Food Development Center’s data reveals that the annual sales of green food escalated to 507.57 billion yuan in 2020, denoting a notable 9 percent increase compared to the preceding year.

However, when considering the production aspect, it is important to note that the production of green food often entails the utilization of additional labor and organic fertilizers. While this approach contributes to the enhancement of environmental sustainability, it can potentially lead to a reduction in agricultural product yield. Furthermore, it inevitably raises the cost for producers. The higher cost and sales price associated with green food production present challenges in meeting the market demand due to the economic implications [7, 8]. The discrepancy between market demand and supply has exerted a profound impact on the efficacy of scale and the competitive advantage of the green food industry, thus posing formidable challenges to the sustainable development of said industry. Consequently, the effective resolution of the market demand-supply dichotomy in the realm of green food, coupled with the augmentation of market share and profitability within the industry, stands as an imperative subject necessitating thorough examination at present.

In the contemporary era marked by rapid shifts in consumption patterns, the influence of digitalization on consumer behavior has gained heightened prominence. This trend has been especially accentuated by the outbreak of the novel coronavirus pneumonia (COVID-19) pandemic, which has led to a broader and deeper integration of the Internet into people’s daily lives. Internet penetration refers to the widespread adoption and application of internet technology in various societal domains. This phenomenon not only revolutionizes information dissemination and transaction methods but also exhibits substantial potential in fostering sustainable consumption [9], propelling the transformation of green manufacturing industries [10], and mitigating environmental pollution [11]. This phenomenon not only revolutionizes the modes by which information is disseminated and commercial transactions occur, but also engenders positive effects in fostering environmentally conscious patterns of consumption, driving the shift towards ecologically sustainable manufacturing practices, and curbing the deleterious impacts of environmental pollution. Extensive research conducted thus far substantiates the positive impact of Internet penetration on facilitating the development of sustainable industries and enhancing the ecological environment. Regrettably, the academic community has not allocated adequate attention to the challenges faced by the green food industry in terms of its lagging development, as well as the potential role that Internet penetration might play in addressing these issues. As a result, further exploration is warranted to determine whether Internet penetration can effectively promote the advancement of the green food industry. Against this backdrop, the primary objective of this paper is to comprehensively investigate the influence of prices and the Internet on the development of the green food industry, encompassing both the supply and demand aspects. Moreover, an essential aim is to explore the role played by the penetration and diffusion of the Internet in shaping the impact of prices on the progression of the green food industry. It is noteworthy to highlight that previous scholarly inquiries have predominantly relied on qualitative or econometric analysis methods for data processing and evaluating the factors that impact the green food industry. In contrast, the present paper adopts a distinctive approach by employing a system dynamics model to specifically examine the alterations in industry dynamics triggered by price and Internet-related factors within the green food industry. This research methodology enables the identification of potentially effective solutions that can contribute to the sustainable development of the green food industry.

The subsequent sections of this paper are structured as follows: Section 2 presents a comprehensive literature review and outlines the research hypotheses derived from the existing body of scholarly work. Section 3 introduces the methodology employed in this study, including the utilization of a causal feedback diagram and a stock flow model to analyze the interrelationships within the green food industry. Section 4 delineates the data sources employed and expounds upon the outcomes derived from model testing and simulation analysis under various scenarios. The concluding section offers a thorough discussion of the main findings, summarizes the key insights, and highlights the limitations inherent in this study.

## Literature review

### Reviews of the impact of the price on the development of the green food industry

Prior research in the realm of traditional food studies has consistently demonstrated that individuals exhibit a pronounced sensitivity to price when it comes to general food consumption [12] Fluctuations in food prices exert direct ramifications on household consumption patterns, individuals’ subjective well-being, and overall household welfare [13–15]. To be specific, a mere one percent increase in food prices has been found to diminish household benefits by a substantial 21.3 percent [16] Given its necessitated adherence to stringent supervision throughout the production process, green food incurs considerably higher total costs compared to traditional food. While green food cultivation generates increased income for agricultural producers, it also entails higher purchase prices for consumers [17] Scholars have offered diverse viewpoints regarding the influence of green food prices on consumers. On one hand, certain researchers have examined consumer intentions and discovered that individuals are willing to pay premium prices for green foods. These studies propose that agricultural products featuring geographical indication (GI) labels can effectively diminish consumers’ price sensitivity [18]. Green food is commonly regarded as a symbol of exceptional quality, healthiness, and environmental friendliness. Such positive perceptions prompt certain consumers to accept a certain premium for green foods [19, 20].

On the other hand, other scholars have explored the purchasing behavior associated with green foods and have presented findings indicating that the elevated prices of green foods could potentially deter a larger portion of the population from purchasing them. However, it is noteworthy that some researchers have also highlighted that despite the positive recognition of green foods, the high prices might exceed the budget limitations and willingness to pay of many consumers. In a recent empirical survey, it was discovered that a substantial proportion of consumers (approximately 90%) expressed their willingness to allocate household funds towards purchasing certified products. Nevertheless, the study also revealed a significant disparity between consumers’ expressed intentions and their actual purchasing behavior, as only 27.57% of consumers were willing to translate their intentions into tangible actions. Additionally, it is noteworthy that among the consumers willing to make a purchase, 72% of them were unwilling to bear a premium of more than 20% for certified products [21]. Another survey revealed that as the premium attached to environmentally friendly products rises, consumer demand becomes more responsive to price fluctuations [22]. Furthermore, consumers tend to overestimate their actual expenditures on green foods [23]. This heightened sensitivity to price fluctuations can be attributed, in part, to the existing disparity between consumer attitudes, purchase intentions, and actual behavioral patterns regarding the consumption of green foods. It is noteworthy that price has emerged as a significant barrier hindering the widespread adoption of sustainable consumption practices in the green food sector [24–26].

### Reviews of the relationship between the Internet and the consumption of green food

The Internet assumes a pivotal role in enhancing the quality of individual consumption, and its impact has led to certain improvements in population consumption patterns. Moreover, the Internet has indirectly fostered technological innovation and enhanced product quality among green food suppliers [27]. Being the most commonly utilized information channel during the process of seeking food risk information [6], the development and utilization of the Internet can positively contribute to the overall advancement of household consumption [28, 29]. The open and interactive nature of the Internet platform serves to effectively stimulate individual consumption enthusiasm and initiative. Additionally, it facilitates connections among various market players and the government, resulting in reduced transaction costs and enhanced transaction facilitation between buyers and sellers [30]. Consequently, these developments significantly elevate consumer utility and welfare [31]. E-commerce, facilitated by the Internet platform, also proves effective in meeting consumer demands, mitigating supply-demand conflicts, and driving consumption growth and the upgrading of consumption patterns [32]. The widespread availability of online marketing platforms has further expedited the transformation of consumer consumption patterns, where personalization, quality, experience, and emotionality have become increasingly prominent features [33]. Furthermore, it has been observed that the adoption of the Internet is significantly and positively correlated with food consumption, thereby exerting a positive influence on consumption levels, giving rise to a notable demand-side revolution [34, 35].

Green food, being recognized as a healthy and environmentally friendly product, possesses unique personalized and emotional attributes. The development of the Internet has, to a certain extent, played a role in facilitating the sustained growth of green food consumption [36]. Firstly, the Internet serves as a platform for disseminating comprehensive and up-to-date information about green foods to a broad audience. Consumers can access detailed information about green food production practices, certification processes, nutritional value, and environmental impact. This transparency enhances consumer awareness and knowledge, enabling them to make informed choices regarding green food consumption. Secondly, the Internet facilitates direct communication between green food producers and consumers. Online platforms allow consumers to interact with producers, ask questions, and receive personalized recommendations. This direct interaction fosters trust and strengthens the relationship between consumers and green food producers, creating a sense of community and fostering consumer loyalty. Thirdly, e-commerce platforms and online marketplaces provide convenient access to a wide range of green foods. By allowing consumers to browse, compare prices, and make purchases online, these platforms remove geographic barriers and expand market reach. This enhanced accessibility and convenience encourage continued green food consumption. Moreover, from the merchant’s standpoint, the Internet plays a pivotal role in enabling merchants to utilize digital marketing strategies for effective promotion of green foods. Through targeted advertising, impactful partnerships, and online campaigns, merchants can raise consumer awareness, generate interest, and stimulate consumer demand for green foods. Leveraging Internet-based marketing techniques, merchants can precisely target specific consumer groups interested in sustainable and healthy food choices. This approach allows for the maximization of promotional efforts and significantly enhances the impact of marketing initiatives.

#### The research gap

In previous studies examining the relationship between price and the green food industry, most researchers have primarily focused on consumer behavior and consumption patterns at the end of the green food industry chain. Specifically, they have investigated the influence of price on consumers’ purchase intentions and behaviors related to green food. These studies have indicated that consumers may express a willingness to accept premium prices for green food, but this willingness does not necessarily translate into actual consumption behavior. Consequently, higher price levels may lead to a lack of effective demand for green food. Furthermore, scholars have acknowledged the significant impact of Internet penetration on both consumer behavior and merchants’ marketing strategies. However, limited attention has been given to the question of how price fluctuations and Internet penetration can influence the development of the green food industry through consumer behavior, and the specific mechanisms by which this influence is achieved. Thus, the objective of this paper is to explore the impact of price volatility and Internet penetration on the green food industry, focusing on the effective connection between supply and demand. By utilizing a System Dynamics (SD) model, this research aims to analyze the mechanisms and processes involved. This approach not only expands the scope of existing research but also provides a novel research method to gain insights into the dynamics of the green food industry.

## Model development

The concept of system dynamics (SD) was introduced by Professor Forrester of MIT in 1956, stating that these thinking and analysis methods can better solve the dynamic complexity problem [37]. The SD model is often used in strategy analysis, and is composed of causal feedback, stocks, flows, and auxiliary variables. When building the SD model, the above elements are defined and checked first. Second, the simulation is performed based on the initial values of the data within a reasonable period. Third, the model is validated by comparing the output values of the simulation with past data. Finally, a series of scenario analyses of the changes in the key parameters are performed, to examine the simulation results of the system [38]. System dynamics models have gained widespread utilization in various fields of study, including market behavior [39], economic growth [40], and policy evaluation [41]. Notably, some scholars have successfully applied system dynamics models to examine the influencing factors of energy consumption in the food industry [38], milk production [42], and drivers of sustainable development in marine fisheries [43] within different industries. These studies have successfully depicted the trends in industrial development and the effects of each factor within the causal chain on the respective industries, providing valuable insights into their dynamics.

The green food industry evolved from the food industry and added more distinctive attributes while retaining traditional industry characteristics. The development of the green food industry can bring higher utility and efficiency. The green food industry contains supply and demand subsystems. There are numerous complex relationships within the two subsystems, which have obvious nonlinear and dynamic characteristics. The SD model can fully describe the dynamic causal mechanism within the system and link the past to the present by showing how the current situation arises and then extend the present to a persuasive future based on a variety of strategic options [44]. For these reasons, this paper uses the SD method to analyze the system behavior of the green food industry and focuses on the impact of price and Internet penetration on the green food industry from a dynamic perspective.

### Causal feedback diagram of the green food industry

**Fig 1:**
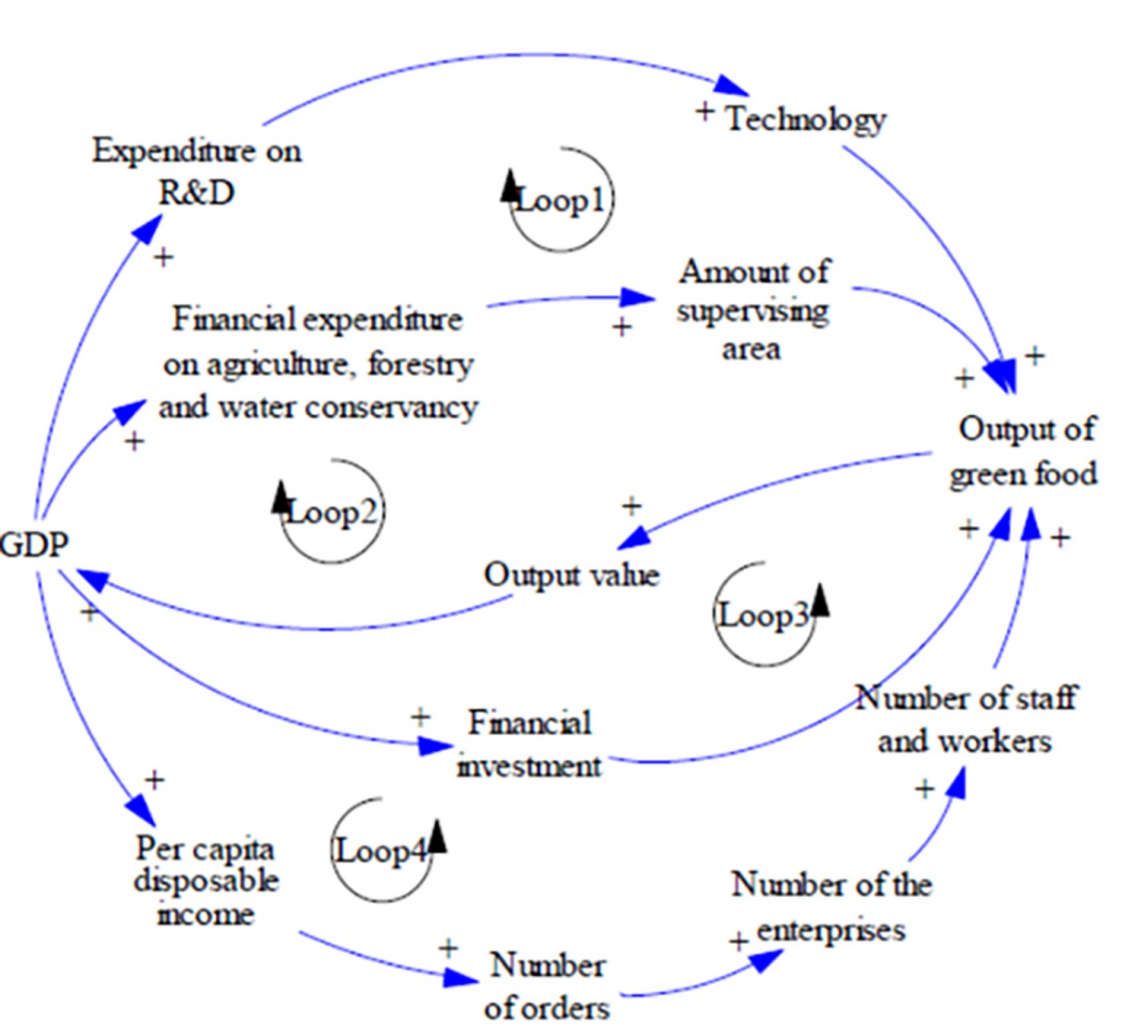
Causal feedback diagram of the green food industry.

The causal feedback diagram serves as a comprehensive representation of the principal variables and their interconnected feedback relationships within the object system. The construction of a causal feedback diagram forms the foundational step towards the establishment of a stock-and-flow modeling framework. In the case of the green food industry system, the system dynamics (SD) model is divided into two distinct subsystems, namely supply and demand. As depicted in (Fig 1), the model encompasses four positive feedback loops. Within the supply subsystem, the production of green food is influenced by four key factors: the geographical area of origin, financial investment, the size of the workforce, and technological advancements. Increased production of green food leads to a corresponding escalation in its output value, consequently fostering the growth of the gross domestic product (GDP). Conversely, the demand subsystem exhibits an interdependency with the GDP. As the GDP experiences an upswing, personal income levels also rise, thereby stimulating an increase in individual consumption patterns. Consequently, the demand for green food experiences a commensurate augmentation. The heightened demand acts as a catalyst, inducing numerous enterprises to enter the green food industry, thereby amplifying the labor demand within the sector. The surge in labor participation subsequently translates into augmented green food output and output value, thereby contributing to an overall rise in the GDP. By scrutinizing the cause-and-effect relationships, it becomes evident that the supply and demand subsystems within the green food industry system exert mutual influence and operate in tandem. This symbiotic relationship culminates in an expansion of the green food output value, which ultimately propels the growth and advancement of the green food industry.

### Stock-and-flow model of the green food industry

**Fig 2:**
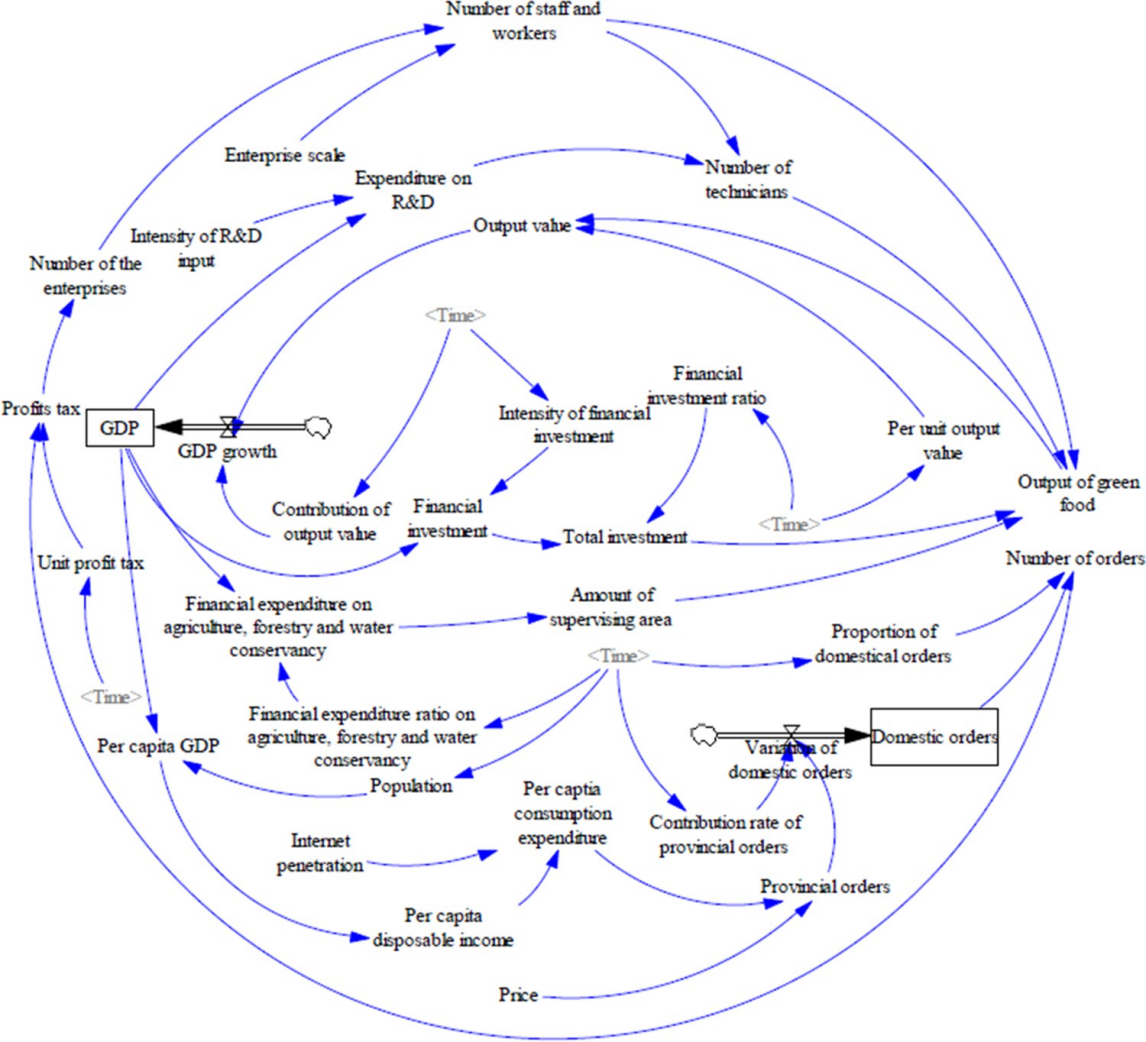
Stock-and-flow model of the green food industry.

The primary purpose of the causal feedback diagram is to depict the causal relationships among the factors within the green food industry system. While this diagram provides a visual representation of the system’s structural connections, it does not quantify the magnitude of influence exerted by each factor on the development of the green food industry. The construction of a stock-and-flow model, based on the causal feedback, is crucial in order to develop a comprehensive understanding of the dynamics of the green food industry system. This model should adhere to the requirements set forth by the system dynamics framework, while also taking into account the specific characteristics and circumstances of the green food industry, with the overarching goal of fostering sustainable development within the industry (Fig 2). The stock-and-flow model encompasses both logical and mathematical relationships among the variables, which are classified into four distinct types: stocks, rates, auxiliaries, and constants. Within the system dynamics model of the green food industry, GDP and domestic orders serve as stocks (with rates accounting for GDP growth and variations in domestic orders), while each subsystem incorporates corresponding auxiliaries and constants as influencing factors. In this study, the output value is employed as an observational variable to gauge the level of development within the green food industry. Additionally, price and Internet penetration are examined as variables to analyze their respective impacts on the green food industry. Moreover, the study also explores the role of Internet penetration in influencing the relationship between price and the green food industry.

## Case study

### Case background and data resource

This study focuses on Heilongjiang Province as a case study, situated in the northeastern region of China, renowned as a significant hub for commodity grain production within the country. In 2019, the crop sowing area in Heilongjiang Province amounted to 14.77 million hectares, constituting 8.9% of the national sowing area. The favorable geographical conditions in the province provide a robust foundation for the advancement of the green food industry. Moreover, Heilongjiang Province has played a pioneering role in the establishment and progression of the green food industry, boasting numerous accomplishments in market development and brand building. Consequently, valuable experiences have been accumulated, contributing to the overall development of the national green food industry. In 2020, Heilongjiang Province standardized 15 production bases, accounting for 20.2% of the national total. These standardized production bases encompassed an area of 4.183 million hectares, accounting for 36.8% of the country’s total, consistently securing the province’s position as a leader in this regard. Recognizing its significance, the green food industry was included among the four strategic industries prioritized by the Heilongjiang provincial government in 2019. Accordingly, plans were formulated to define the direction and developmental focus of the industry. The development of the green food industry in Heilongjiang Province not only contributes to the preservation of the ecological environment and the enhancement of food quality but also fosters high expectations for the prosperity of the rural economy and the revitalization of rural areas. This multifaceted impact holds immense significance for achieving high-quality and sustainable regional development.

To obtain the necessary research data, official documents from Heilongjiang Province are utilized, particularly for variables such as GDP and population. Additionally, green food orders are directly sourced from the Statistical Yearbook of Heilongjiang spanning the period from 2000 to 2021. Certain constant values, such as the proportion of domestic orders and the financial investment ratio, are derived through calculations based on relevant data. The table function is employed to handle constants that exhibit noticeable fluctuations. By inputting historical data for specific years, the model is enhanced to better simulate real-world conditions. Furthermore, some auxiliary variables are derived from the outcomes of econometric regressions. These variables include green food output, per capita consumption expenditure, and provincial order. The utilization of econometric regressions allows for a more comprehensive understanding of the relationships and dynamics among these variables within the model. Table 1 presents the descriptive statistics of the key variables under consideration. Due to data availability limitations, the Internet Penetration variable is restricted to the period from 2003 to 2021. Before 2015, the ratio of the number of Internet users to the total population in Heilongjiang Province is utilized, while after 2016, the ratio of the number of mobile Internet users to the total population is employed. This choice reflects the rapid growth of the mobile Internet sector in China in recent years. However, it should be noted that data for Expenditure on R&D, Output value of green food, and Profits tax in 2021 are currently unavailable. As a result, the data for these three variables are limited to the year range of 2020 and prior. The majority of the regressions conducted in this study employ linear regression techniques, yielding goodness-of-fit measures exceeding 90 percent. These regressions enable a quantitative assessment of the relationships among the variables under investigation, providing valuable insights into their dynamics within the system dynamics (SD) model. Additionally, in accordance with the rules and principles of the SD model, other variables are defined and set. These variables play crucial roles in capturing the interdependencies and feedback mechanisms within the green food industry system. For the purposes of this paper, the simulation time span is established from 2000 to 2030, with a yearly time step. This extended time horizon allows for an examination of the long-term dynamics and trends within the green food industry system, providing valuable insights into its future trajectory.

**Table 1:** Descriptive statistics.

Variable	Obs	Mean	Std. dev.	Min	Max
GDP	22	8705.05	4053.216	2855.5	14858.2
Internet Penetration	19	0.422	0.312	0.059	0.954
Population	22	3642.518	240.778	3125	3833.4
Expenditure on R&D	21	101.452	55.930	14.9	173.2
Financial expenditure on agriculture, forestry and water conservancy	22	393.953	337.364	28	914.53
Per capita disposable income	22	17010.77	9599.626	4912.9	33646
Per captia consumption expenditure	22	12300.17	6642.663	3824.4	24422
Amount of supervising area	22	5440.873	2602.744	618	8816.8
Number of the enterprises	22	566.682	327.666	82	1158
Number of staff and workers	22	14.94091	7.622	3.3	24.9
Total investment	22	143.777	101.550	14.4	343.8
Output value of green food	21	670.379	629.809	24.2	1650
Profits tax	21	47.445	35.797	4.7	98.9
Total orders	22	484.929	309.517	52.5	965
Provincial orders	22	126.436	58.738	16.2	214.3
Domestic orders	22	460.886	299.081	42	924.9

### Model test

The assessment of the SD model primarily comprises four key elements, namely extreme tests, sensitivity tests, structural tests, and validity tests. The fundamental objective behind conducting model testing is to ascertain the accuracy of the model in replicating real-world systems. Within the scope of this paper, the model underwent rigorous examination through sensitivity tests, structural tests, and validity tests.

#### Sensitivity test

**Fig 3:**
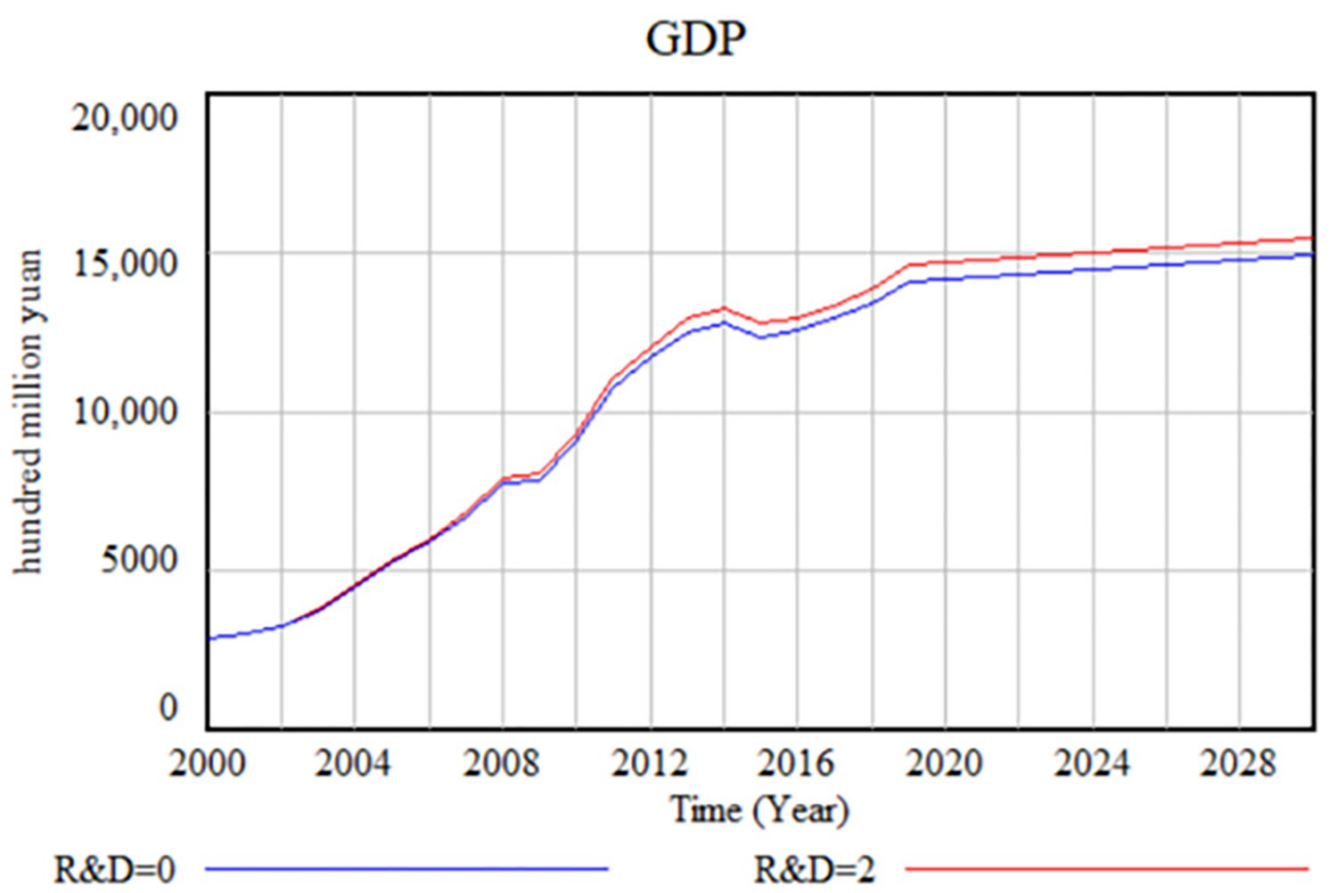
Sensitivity test of SD model.

The sensitivity test serves to evaluate the robustness of the model by investigating whether other variables respond reasonably when a single variable is modified. Essentially, it examines whether altering the value of one variable causes the remaining variables to adjust solely in terms of their values without affecting the system’s behavior. If the SD model exhibits behavioral sensitivity, its suitability for simulation purposes becomes questionable. Within the scope of this paper, representative variables, such as the intensity of R&D input, were carefully selected for sensitivity testing. To initiate the sensitivity test, the intensity of R&D input was systematically varied, ranging from the minimum value of 0 to a value of 2. The simulation was executed sequentially, enabling a comparison between the two scenarios to ascertain the presence of behavioral sensitivity in the model. (Fig 3) provides a visual representation of the results, indicating that as the R&D intensity value is adjusted from small to large, GDP correspondingly increases while maintaining a consistent developmental trend of continuous growth. Subsequently, all stock changes were thoroughly examined, and no instances of behavioral sensitivities were observed. Although the sensitivity test aids in identifying crucial influencing factors, it alone does not provide an indication of their magnitudes of impact. Consequently, quantification through multiple scenario simulations becomes imperative to gauge the extent of their effects.

#### Structural test

**Fig 4:**
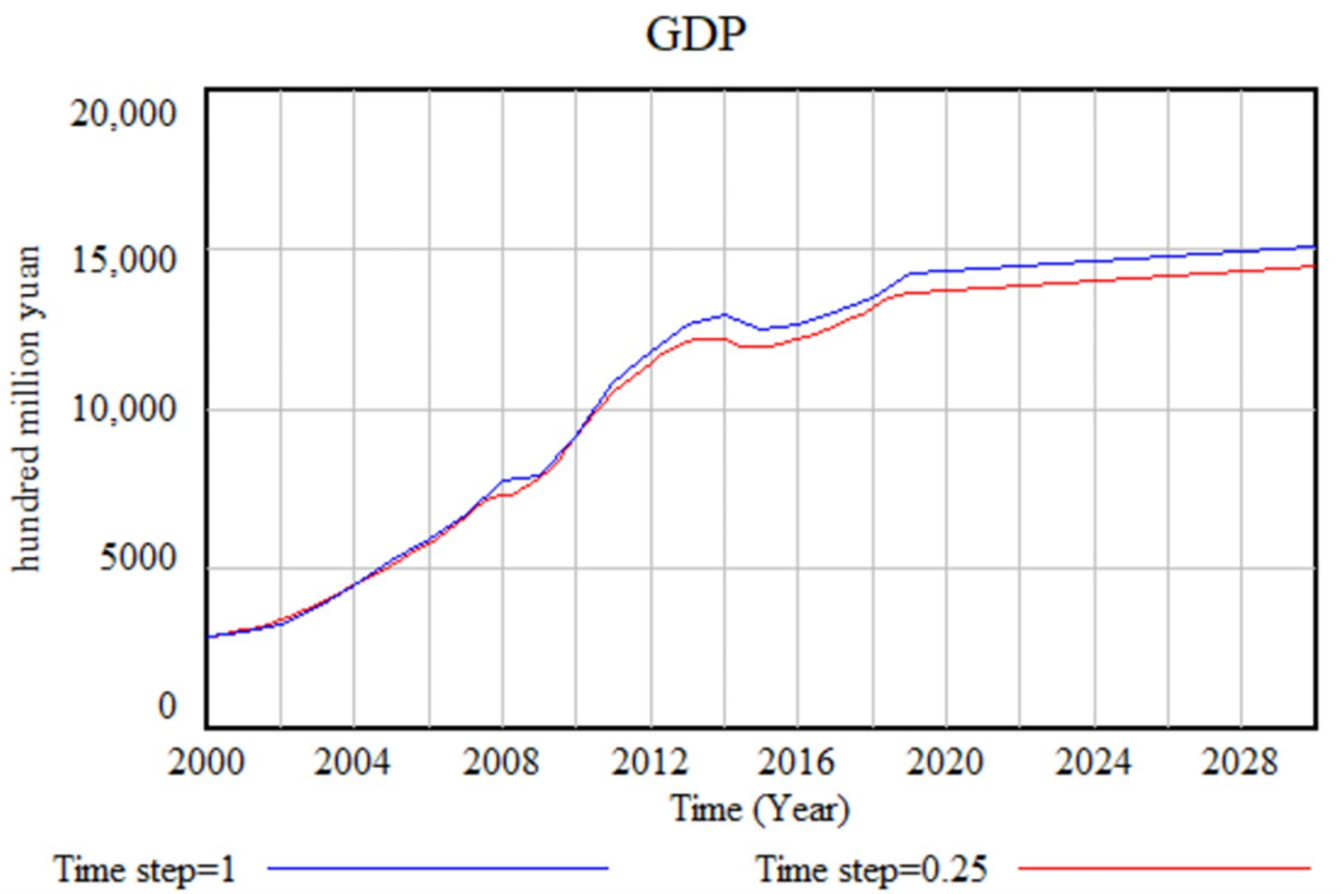
Structural test of SD model.

The structural test aims to examine whether the simulation results are influenced by the time step utilized in the SD model. Since the SD model is founded on calculus principles, conducting a time step test becomes essential. The outcome of this test is illustrated in (Fig 4), where the simulation step is reduced from 1 year to 0.25 years. It is noteworthy that the simulation results demonstrate no significant changes, indicating that the time step employed does not exert a substantial impact on the model’s simulation outcomes.

#### Validity test

The validity test serves to assess the effectiveness of the model by examining the disparity between simulated and historical values. In order to explore the validity of the model, representative variables with available historical data, such as GDP and domestic orders, were meticulously selected. The findings presented in Table 2 indicate that the deviation between the actual and simulated values of GDP and domestic orders from 2010 to 2021 is below 10 percent. Consequently, it can be inferred that the green food industry system dynamics model exhibits a high degree of accuracy in fitting the data. The small margin of error between the simulation and the actual values falls within an acceptable range. These results validate the model’s capability to effectively mirror the functioning of the real system.

**Table 2:** Error rate of the main variable.

Year	GDP			Domestic orders		
	Actual value	Simulation value	Error rate	Actual value	Simulation value	Error rate
2010	8308.3	9122.69	0.098	305.76	321.76	0.05
2011	9935.0	10773.70	0.084	363.50	376.85	0.04
2012	11015.8	11780.40	0.069	389.50	404.65	0.04
2013	11849.1	12623.90	0.065	478.90	493.73	0.03
2014	12170.0	12932.40	0.063	554.10	564.90	0.02
2015	11690.0	12469.10	0.067	567.00	579.50	0.02
2016	11895.0	12666.90	0.065	890.40	842.01	-0.05
2017	12313.0	13029.30	0.058	906.60	855.11	-0.06
2018	12846.5	13504.90	0.051	924.90	869.56	-0.06
2019	13612.7	14228.70	0.045	876.20	884.76	0.01
2020	13633.4	14304.60	0.049	882.70	901.22	0.02
2021	14858.2	14380.80	-0.032	898.80	917.81	0.02

### Parameter setting

This study employs multiple scenario simulations to analyze the influence of price and Internet penetration on the green food industry. In the supply subsystem, the output calculation relies on the estimation of the C-D production function. Meanwhile, the demand subsystem utilizes regression analysis to determine per capita consumption expenditure, considering two variables: per capita income and Internet penetration. Internet penetration is measured using the proportion of Internet users in Heilongjiang Province from 2000 to 2021 The green food order is affected by both consumption levels and prices. The equation for assessing the impact of price on green food order is derived from linear programming, building upon previous research on residents’ willingness to pay a premium [45]. To investigate the effects of price and Internet penetration on the development of the green food industry, this study designs simulation scenarios with varying factor values. Table 3 presents the simulation scenarios, where scenarios 1 and 2 involve a 20 percent and 40 percent increase in prices, respectively. Scenario 3 examines the impact of a 20 percent increase in Internet penetration. Scenarios 4 to 7 explore the combined role of price and Internet penetration by varying their percentages of increase, shedding light on the influence of the Internet in the context of price effects on the green food industry.

**Table 3:** Design of the simulation scenarios.

Scenario	Price	Internet penetration
Basic value	1	0.23
Scenario 1	1.2	0.23
Scenario 2	1.4	0.23
Scenario 3	1	0.43
Scenario 4	1.2	0.43
Scenario 5	1.2	0.63
Scenario 6	1.4	0.63
Scenario 7	1.4	0.83

### Simulation results

**Fig 5:**
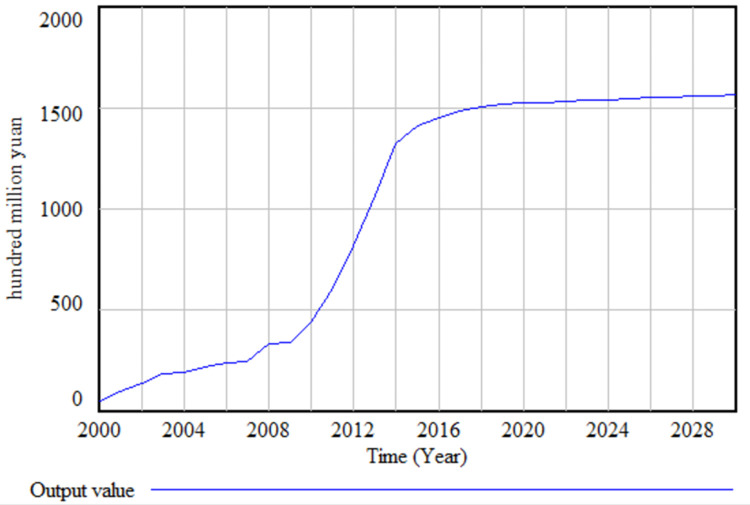
Simulation results of green food output value.

The simulation results depicted in (Fig 5) reveal an "S" shaped growth pattern for the green food industry from the year 2000 to 2030. The growth rate of the green food output value demonstrates a gradual deceleration starting from 2014. By 2019, the green food industry transitions into a phase of slow and stable growth. This observation suggests that the industry has reached a mature stage with a relatively steady growth trajectory.

**Fig 6:**
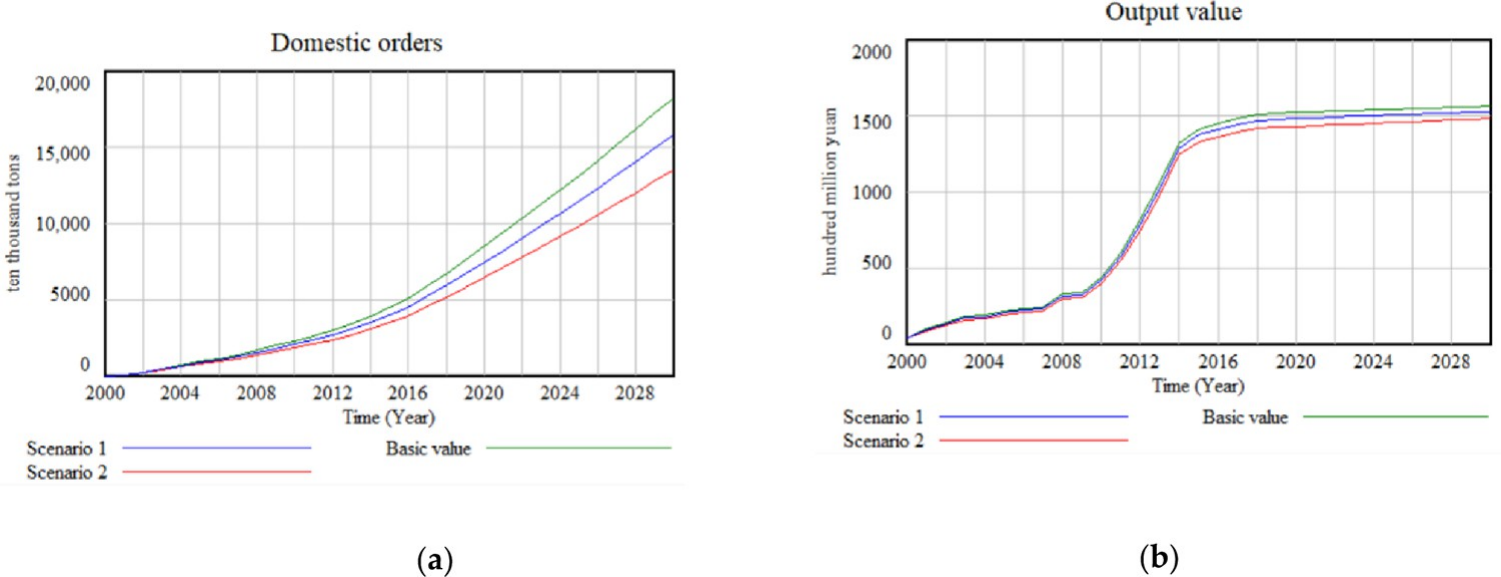
Simulation results for different prices: (a) Domestic orders when the green food price increases by 20% to 40%; (b) Output value when the green food price increases by 20% to 40%.

To test the validity of Hypothesis 61, simulations are conducted for Scenario 1 and Scenario 2. The simulation results demonstrate a decrease in the accumulation of domestic orders in tandem with the increase in prices. As the green food industry expands, the impact of prices on the demand for green food becomes increasingly significant, indirectly constraining the growth of green food output value. (Fig 6) illustrates that as the price of green food increases by 20 percent to 40 percent, both the orders and the output value of green food exhibit a continuous decline. However, the output value experiences a more pronounced decrease compared to the decline in orders. Further analysis reveals that the price elasticity of demand for green food evolves from 0.5 in 2000 to 0.7 in 2030 when prices increase by 40 percent, assuming other factors remain constant. The sensitivity of consumers to changes in green food prices increases slightly over time in the case of higher premiums for green food. Therefore, we can argue that price increases induce changes in other factors that indirectly contribute to a decline in the value of green food production.

**Fig 7:**
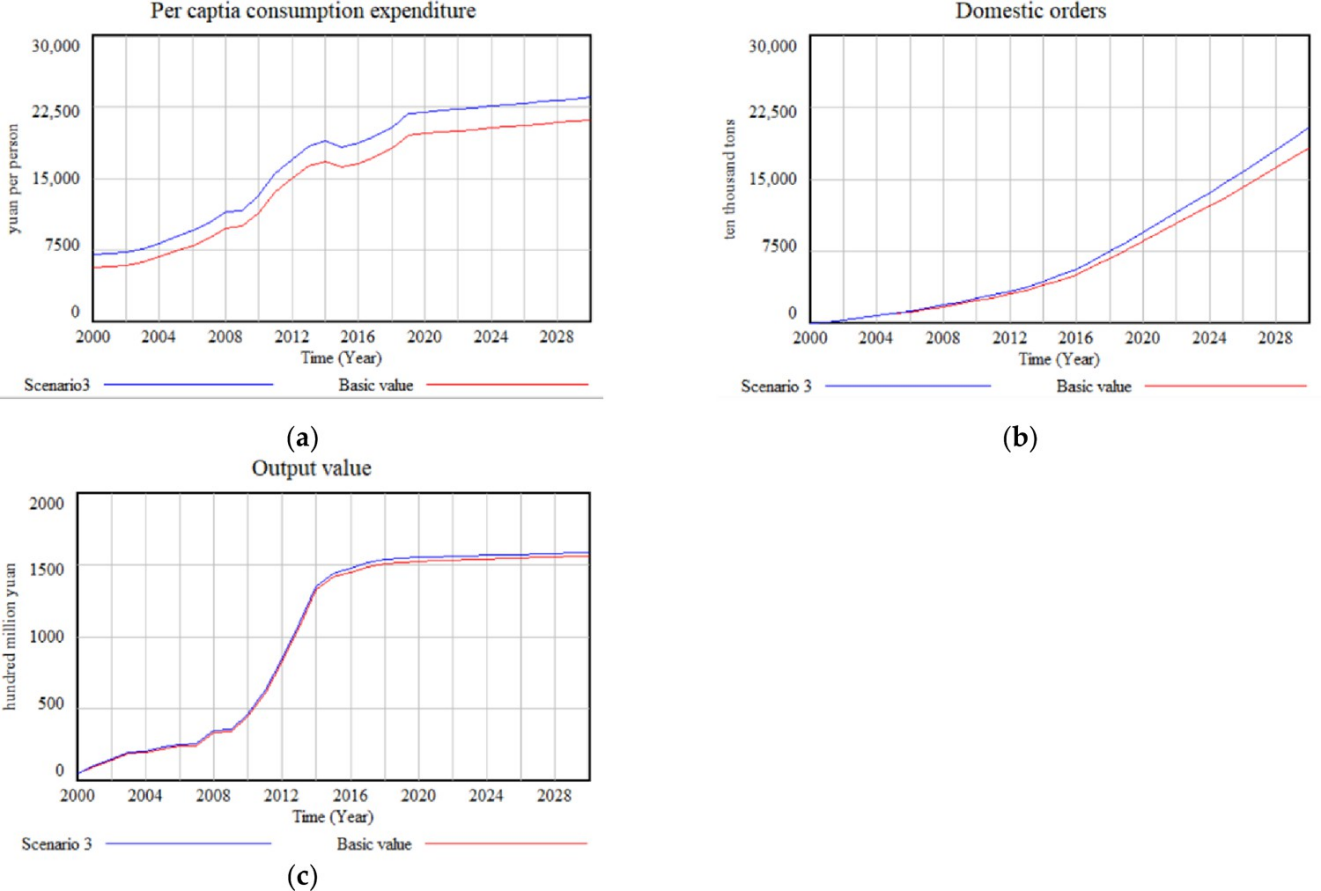
Simulation results for different Internet penetration: (a) Per capita consumption expenditure of a 20% increase of internet penetration; (b) Domestic orders of a 20% increase of internet penetration; (c) The output value of a 20% increase of internet penetration.

Based on the findings, it can be concluded that Hypothesis 1 is valid. As the green food industry progresses, the volatility of demand caused by fluctuations in green food prices increases, and price increases impose limitations on the industry’s development. Furthermore, Hypothesis 2 is also supported by the simulation results. In Scenario 3, where Internet penetration rate is adjusted and simulated (Fig 7), residents’ consumption levels increase due to the expanded Internet penetration. Consequently, the demand for green food rises correspondingly, ultimately fostering the development of the green food industry. The single-factor simulation analysis demonstrates that there are distinct variations in the effects of price and Internet penetration on the green food industry at different stages. Notably, the inhibitory impact of price on the industry’s development is more substantial than the promoting effect of Internet penetration.

**Fig 8:**
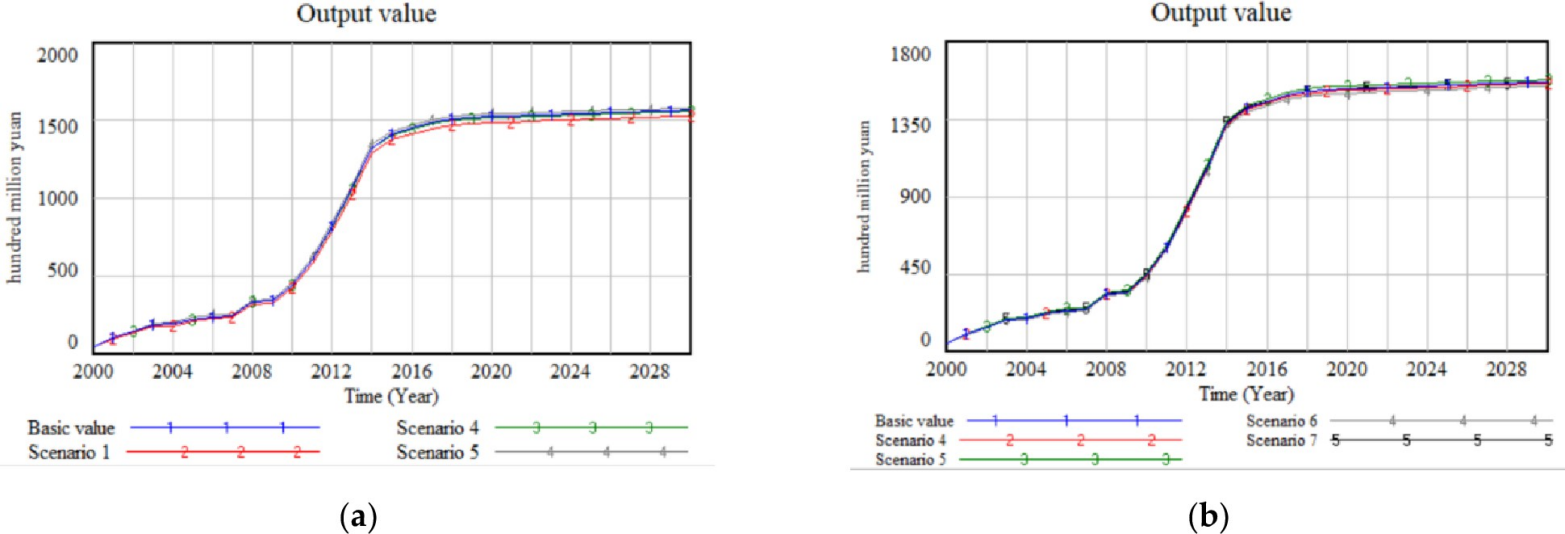
Simulation results of the combined scenario: (a) Output value when the internet penetration increases by 20% to 40%; (b) Output value when prices and Internet penetration increase at different rates.

In the simulations of combined scenarios, the results demonstrate that Internet penetration plays a crucial moderating role in mitigating the adverse impact of premiums on the development of the green food industry, thus validating Hypothesis 3. Comparing the combined scenarios with the single-factor scenario reveals that Internet penetration plays a more significant role when it comes to price increases compared to scenarios with constant prices (Fig 8). The results of the comparison among all the combined scenarios indicate that the value of green food production, in descending order, is scenario 5, scenario 7, baseline value, scenario 4, and scenario 6. Notably, the output value of green food in scenario 5 exceeds that of scenario 4 and the baseline value. This signifies that the widespread adoption of the Internet can effectively mitigate the negative impact of prices on the green food industry. Furthermore, the comparison between scenario 6 and scenario 7 further confirms this conclusion. The results obtained by comparing the output value of scenario 4 with that of scenario 6 demonstrate that scenario 6 yields a lower output value than scenario 4 and the baseline value. This implies that the inclusion of the Internet factor in the system can alleviate the inhibitory effect of prices on the development of the green food industry. However, it is important to note that the effect of Internet penetration on the industry is still relatively smaller than the effect of prices.

## Discussion

The development of the green food industry is a complex and multifaceted process characterized by the simultaneous growth of supply and demand. In the green food industry system, the demand side stimulates the supply side, and vice versa, creating a positive feedback loop that drives the continuous development of the industry in a favorable direction. This study has successfully established and simulated a dynamic model of the green food industry system. The simulation results reveal that the green food industry in Heilongjiang Province has experienced a transition from slow growth to rapid growth since 2000. Similar to many other industries, the green food industry also follows an "S"-shaped growth trend. After a preliminary analysis, it can be inferred that the current green food industry has reached a mature stage and is exhibiting a relatively long tail, as depicted in (Fig 5). This exploration is essential for ensuring the sustainable development of the green food industry in the long run.

### The impact of price on the green food industry

The simulated values of green food output indicate a gradual change in output, with a decrease of 5.6% in 2001 and 2.1% in 2030, given a 20% increase in green food prices. The empirical findings indicate a consistent negative association between price escalation and the progression of the green food industry. However, this suppressive influence exhibits a diminishing trend as the industry evolves. To elucidate further, the observed dynamics can be categorized into three distinct stages. Initially, spanning the years 2001 to 2015, the inhibitory impact of price on green food production experiences a gradual attenuation. Subsequently, from 2015 to 2019, this adverse effect intensifies. Finally, following the threshold of 2019, the inhibitory effect demonstrates a tendency towards stabilization. Undoubtedly, price increments have exerted a substantial constraining influence on the expansion of the green food industry throughout the aforementioned periods. Several reasons can explain this phenomenon. Firstly, during the early stages of the industry’s development, the sales market for green food as an emerging consumer product is not fully established. Secondly, it is noteworthy that consumers’ access to information and their income levels were relatively restricted during these periods. Consequently, the combined effect of market dynamics and consumer attributes contributed to a limited consumer awareness regarding green food. A significant majority of consumers encountered difficulties in embracing premium pricing for seemingly indistinguishable products, thereby resulting in an inadequate effective demand and exerting a pronounced price-suppressive effect on the green food industry [46]. Nevertheless, as a consequence of swift economic progress and enhanced living conditions, individuals have become progressively cognizant of the significance of food safety. This heightened awareness has translated into an exponential surge in the demand for wholesome sustenance and a gradual upsurge in environmental concerns [47]. Consequently, there has been a gradual diminution in the adverse influence of price on the green food industry. Thirdly, the mounting focus of the government on environmental preservation has propelled an augmented supply of green food. Simultaneously, both governmental and corporate endeavors aimed at promoting green food have intensified, leading to an elevation in consumer awareness. However, it is important to acknowledge that certain consumers may find themselves unable to afford the escalating prices, thus impacting the green food industry to varying degrees due to the negative implications associated with price hikes. Post-2019, the green food industry demonstrates a tendency towards maturity, with the impact of a specific range of price premiums on the industry gradually reaching a plateau. During this phase, the green food industry exhibits an adaptive capacity to cope with upward price pressures. Indeed, increased production efficiency, technological innovation, and economies of scale play pivotal roles in reducing production costs and promoting price stability within the green food industry. Such advancements enable consumers to afford higher prices while sustaining their consumption of green foods. Consequently, the negative impact is likely to experience a further decline.

Based on the findings, the following conclusions can be drawn: Firstly, the impact of price increases on the development of the green food industry varies at different stages of its growth. The inhibitory effect of premiums on the industry diminishes as the industry expands. This suggests that price increases have a more substantial limiting effect on the early stages of the industry’s development, whereas their impact becomes relatively less significant as the industry matures. Secondly, as the premium price of green food increases, both the demand and output of green food decrease. However, the decrease in output value is more pronounced compared to the decline in demand. Based on these findings, it is recommended to adjust the premium price of green food within a reasonable range. Particularly, in different stages of the green food industry’s development, sellers should adopt different premium strategies. Furthermore, it is important to acknowledge that green food production entails additional expenses for testing, certification, and management processes, which ultimately contribute to its higher price [48]. In light of this, relevant institutions should provide policy subsidies to environmentally friendly production, reducing the production costs of green food and aligning product prices with consumer demand. As the industry expands and consumer awareness of green food increases, government support for the green food industry can gradually be scaled back, allowing market forces to regulate transactions.

### The role of internet penetration in price influencing the green food industry

The rise of one industry can be attributed to the maturity of another industry, as noted in previous studies [49]. In the context of the new technological revolution, the Internet has permeated various aspects of production and daily life, becoming a crucial driving force for global economic growth and industrial development [50]. The simulation results in this study demonstrate that the demand for green food increases with the growth of Internet penetration. Furthermore, the impact of Internet penetration on the growth of green food demand strengthens over time, while its effect on the growth of green food output weakens. This can be attributed to the green food industry’s inherent public welfare attributes, where government intervention plays a significant role in its development [51]. However, it should be acknowledged that government intervention can also undermine the positive impact of the Internet. Therefore, marketization becomes essential to unlock the full potential of the Internet [52]. This observation further explains why an increase in Internet penetration can significantly boost the demand for green food but does not have a substantial impact on the growth of green food output. Hence, it is crucial to strike a balance between "efficient market" and "efficient government" approaches and recognize the significant role of market allocation. Local governments should minimize unnecessary intervention and establish an enabling institutional environment that allows the Internet to fully and effectively contribute to the green food industry’s development. By combining the strengths of both market mechanisms and government support, a conducive environment can be created to facilitate the sustainable growth of the green food industry in the digital era.

The simulation results within the framework indicate that the Internet can play an effective role in mitigating the negative impact of price fluctuations on the development of the green food industry. As Internet penetration increases, its regulatory effect becomes more pronounced, and there is a dynamic change in this effect over time. When both price and Internet penetration increase by 20 percent, the inhibiting effect of price is slightly stronger than the promoting effect of the Internet. However, as both price and Internet penetration continue to rise, the role of Internet penetration gradually strengthens. Based on these findings, it is concluded that Internet penetration significantly boosts the demand for green food while alleviating the inhibiting effect of prices on the industry’s development, particularly as the industry grows. To leverage this potential, relevant authorities can focus on strengthening Internet infrastructure and promoting the use of Internet applications to enhance the overall Internet penetration rate. Furthermore, green food suppliers can leverage Internet platforms to gain insights into consumer preferences and develop targeted marketing strategies for green food products, ensuring effective fulfillment of consumer needs. By fostering a virtuous cycle of demand and supply through the synergy of the Internet and the green food industry, the sustainable development of the sector can be further promoted.

## Conclusion

The increasing concern of society for environmental protection has brought new opportunities for the development of green food industry in China. By using system dynamics to simulate and analyze the green food industry, we have come to the following conclusions: Firstly, the development of the green food industry also shows an S-shaped trend, and like other industries, it has gone through a phase from rapid growth to steady growth. Secondly, the price increase significantly inhibited the development of green food industry, but the inhibiting effect showed a gradual weakening trend with the growth of the industry. The decline in consumption due to price increases is one of the channels inhibiting the development of the green food industry, but not the most important one. Thirdly, the use of the Internet enhances consumer demand for green foods and mitigates the dampening effect of price increases on the green food industry, an effect that is sustainable. Therefore, the supply side of green food should adjust the premium price within a reasonable range, and the government should also provide appropriate policy subsidies. Using these methods to reduce green food prices and promote the rapid development of the green food industry. In addition, the level of Internet and digital development should be strengthened to enhance the degree of digital integration between the supply and demand sides of green food, and new technological tools should be used to support the sustainable growth of the green food industry.

This study on the dynamic effects of price fluctuations and Internet penetration on the green food industry provides valuable insights, but it also has some limitations that call for further research. First, the study does not delve into the heterogeneity of the effects of price and Internet penetration on the demand for green food among different consumer groups due to data limitations. Exploring how these factors impact diverse consumer segments could yield more nuanced findings. Second, the model focuses on the overall operation of the green food industry system and overlooks individual consumer factors, such as personal consumption habits and subjective intentions, which could influence green food demand. Incorporating these variables would enhance the model’s validity and predictive power. It is important to consider these limitations in future research by integrating individual-level data, conducting surveys or experiments, and exploring additional factors that shape green food demand. Addressing these aspects would provide a more comprehensive understanding of the dynamics between price fluctuations, Internet penetration, and the sustainable production and consumption of green food.

## Supporting information

S1 Appendix (DOCX)

S2 Appendix (XLS)
