# Supplementary material for: Price, internet penetration and green food industry development: Based on the interaction between demand and supply
Source: PLoS One. 2023 Sep 8;18(9):e0289843. doi: 10.1371/journal.pone.0289843 (PMC10490892; doi:10.1371/journal.pone.0289843)
Supplement: S1 Appendix — (DOCX) [file pone.0289843.s001.docx]

**Appendix A**

| Table1 Main variables and parameters of SD model | |
| --- | --- |
| Variable Type | Variable Name(Abbreviation) |
| Stock | Gross Domestic Product (GDP) Domestic orders (DOR) |
| Flow | Incremental Gross Domestic Product (IGDP) Variation of domestic orders (VDOR) |
| Auxiliary | Output of green food (GFP), Output value of green food (GFO), Financial investment (FI), Total investment (TI), Financial expenditure on agriculture, forestry and water conservancy (FEA) , Financial expenditure ratio on agriculture, forestry and water conservancy (FEAO), Amount of supervising area (ASA), per capita GDP (PGDP), Per capita disposable income (PDI), Per captia consumption expenditure (POE), Provincial orders (POR), Number of orders (NOR), Profits tax (PT), Unit profit tax (UPT), Number of the enterprises (NE), Number of staff and workers (NSK), Expenditure on R&D (ERD), Number of technicians (NT), Per unit output value (POV) |
| Constant | Enterprise scale (ES), Intensity of R&D input (IRD), Intensity of financial investment, Internet penetration (IP), Price, Population, Contribution of output value(COV), Proportion of domestical orders (PDOR), Contribution rate of provincial orders (CRPOR), Financial investment ratio (FIR) |

The equations of the dynamic systems model and its original parameters are ascertained through a variety of methodologies, including arithmetic averaging, table functions, and regression analysis. Presented below are the principal equations of the model.

Table2 Equations of main variables

| Type | Variable | Equation |
| --- | --- | --- |
| Stock | GDP | INTEG（GDP+IGDP，2855） |
|  | DOR | INTEG（DOR+VDOR，42） |
| Flow | IGDP | GFP * COV |
|  | VDOR | CRPOR * POR |
| Auxiliary | GFP | EXP(3.845+0.254 * LN(ASA)+0.564 * LN(NT)+0.384 * LN(NSK)+0.015 * LN(TI)) |
|  | GFO | (POV * GFP) |
|  | NSK | EXP(-1.2562+0.826 * LN(NE)+0.477 * LN(ES)) |
|  | TI | FI * FIR |
|  | NT | 0.485+0.096 * NSK+0.001* ERD |
|  | PT | UPT * NOR |
|  | NOR | DO/PDOR |
|  | POR | EXP( 0.2386 * (LN(POE))^2 - 3.6626 * LN(POE) + 18.149) * (1.6-0.6 * Price) |
|  | POE | 1073.96+0.5395 * PDI+7036.52 * IP |
